# Efficacy of Ozone Bagging Therapy in Equine Chronic Distal Limb Wounds: Clinical Evaluation of Eight Cases

**DOI:** 10.3390/vetsci13010016

**Published:** 2025-12-23

**Authors:** Călin Cosmin Repciuc, Nicușor-Valentin Oros, Ștefana Maria Cristina Mureșan, Bogdan Sevastre, Jean Guilherme Fernandes Joaquim, Liviu-Ioan Oana

**Affiliations:** 1 Department of Surgery, Faculty of Veterinary Medicine, University of Agricultural Sciences and Veterinary Medicine, 400372 Cluj-Napoca, Romania; calin-cosmin.repciuc@usamvcluj.ro (C.C.R.); liviu.oana@usamvcluj.ro (L.-I.O.); 2 Department of Internal Medicine, Faculty of Veterinary Medicine, University of Agricultural Sciences and Veterinary Medicine, 400372 Cluj-Napoca, Romania; nicusor-valentin.oros@usamvcluj.ro; 3 Department of Pathophysiology, Faculty of Veterinary Medicine, University of Agricultural Sciences and Veterinary Medicine, 400372 Cluj-Napoca, Romania; bogdan.sevastre@usamvcluj.ro; 4 Bioethicus Institute, Botucatu 18605-545, SP, Brazil; dr.jeanjoaquim@gmail.com

**Keywords:** horse, distal limb, ozone therapy, chronic wounds, wound healing

## Abstract

Limb wounds in horses represent a significant therapeutic challenge due to poor vascularization, reduced skin elasticity, and high risk of complications such as exuberant granulation tissue. Conventional treatments sometimes fail to provide satisfactory healing outcomes, leading to prolonged recovery and increased costs. This study aimed to evaluate the efficacy of topical ozone therapy using the bagging method in promoting the epithelialization and contraction of chronic distal limb wounds in horses refractory to conventional management. Eight horses, aged 3–21 years, with chronic wounds averaging 48.79 ± 21.20 cm^2^, were treated exclusively with ozone (50 μg/mL) administered by bagging for 30 min every 48 h until complete healing. All cases achieved full wound closure within 27–91 days without systemic medication or major complications. Macroscopic evaluation showed favorable healing, with the restoration of skin pigmentation and hair growth in most cases, while only minimal fibrous scarring was observed in a few patients. Statistical analysis revealed significant improvements in epithelialization, particularly during the last four weeks of treatment. These findings suggest that ozone bagging therapy is a simple, cost-effective, and well-tolerated method that may enhance the healing of chronic distal limb wounds in horses. Further controlled trials are needed to standardize treatment protocols and compare ozone with conventional therapies.

## Simple Summary

Wounds on a horse’s limbs sometimes take a long time to heal and can easily develop complications such as proud flesh (exuberant granulation tissue) or scars that are painful and difficult to treat. These complications make recovery frustrating and costly for both owners and veterinarians. Ozone has recently been explored in veterinary medicine because of its ability to fight infection and support tissue repair. In this study, we treated eight horses with long-standing limb wounds that had not healed with traditional therapies. Using a simple technique called the “bagging method,” the affected limb was placed in a sealed bag filled with medical ozone gas for 30 min every two days. All horses recovered completely within two to three months, most with normal skin and hair regrowth and only minimal scarring. Our results suggest that ozone therapy can be an effective and affordable option for managing difficult leg wounds in horses, and it may be worth considering when other treatments fail.

## 1. Introduction

Horses have similar skin features to humans and share common pathologies like exuberant granulating tissue or painful scars, especially at the level of the extremities [1]. Distal limb wounds in horses pose a major clinical challenge due to anatomical and physiological limitations such as reduced vascularization, low muscular support, and a predisposition to exuberant granulation tissue. These factors often result in chronic wounds with delayed epithelialization and poor cosmetic and functional outcomes [1,2,3,4]. Chronic wounds at this level exhibit increased synthesis of collagen but show decreased degranulation of it [4]. Higher and prolonged liberation of profibrotic growth factors and prolonged persistence of elevated fibroblast concentrations are also observed at this level [4]. Limb localized wounds exhibit myofibribroblasts with worse orientation and lead to a lack of contraction and higher scarring rates at the end of the healing process [4].

Chronic wounds are defined as lesions failing to progress through the normal phases of healing within typical timeframe, often due to infection, tissue hypoxia, or persistent inflammation [5]. Several approaches have been used to manage such lesions, including topical antimicrobials, growth factors, negative pressure therapy, and surgical debridement [6,7]. However, the outcomes are inconsistent, and prolonged treatment durations increase both costs and risk of complications.

Ozone therapy has recently gained interest in both human and veterinary medicine for its dual antimicrobial and pro-healing properties [8,9]. At therapeutic concentrations, ozone can support tissue oxygenation, stimulate angiogenesis, and activate local antioxidant mechanisms. Its mild oxidative effect may contribute to bacterial reduction at the wound surface, indirectly creating a cleaner environment favorable for tissue repair [10].

Ozonated oils and water preparations have been reported to improve wound healing and prevent infection [11,12,13]. However, few studies have evaluated the direct exposure of equine wounds to ozone gas. The ozone bagging technique, in which the affected limb is enclosed in an ozone-filled bag, allows for uniform exposure and minimal tissue handling. Initially, higher concentrations are applied to reduce microbial load, followed by lower doses to promote healing following the hormetic effect of this therapy [14,15,16,17].

The present study aims to evaluate the clinical effects of topical ozone applied by the bagging method on chronic distal limb wounds in horses previously unresponsive to conventional therapy. The working hypothesis is that repeated ozone exposure may accelerate epithelialization through both its wound-cleansing and regenerative properties.

## 2. Materials and Methods

### 2.1. Study Design and Subjects

This study was conducted between 2020 and 2024 and included eight adult horses of different sex (three females, five males) aged three to twenty-one years. All horses were presented to the equine clinic of the Faculty of Veterinary Medicine of Cluj-Napoca with chronic distal limb wounds unresponsive to previous conventional treatments received in the field or at other clinics.

Initially, fifteen animals were evaluated; seven were excluded due to either mild superficial lesions (<2 cm^2^), which were expected to heal without prolonged intervention, or owner refusal. Distal lesions were considered ones located below the stifle/elbow. Mild lesions were defined as wounds not requiring extended therapy and without prolonged prior treatment. Chronic wounds were defined as lesions persisting for more than 30 days without measurable contraction or epithelialization despite standard management [1].

### 2.2. Ethical Approval and Consent

Owners provided written informed consent, and this study was approved by the USAMV Cluj-Napoca Ethics Committee (protocol nr. 192/11.02.2020).

### 2.3. Ozone Solution and Administration

Ozone was generated using a Medozon Compact normobaric ozone generator (HERRMANN Apparatebau GMBH, Elsenfeld, Germany) capable of producing concentrations between 2 and 80 μg/mL. All treatments were performed using the bagging technique, following the 2020 Madrid Declaration [10] and WFOT [15] recommendations. Horses remained hospitalized for the duration of the treatment protocol. Materials used included the ozone generator, 10 mL syringes, perfusion tubing, ozone-resistant polyethylene bags, 0.4 μm syringe filters, torniquet bands, transparent plastic foil and millimetric paper.

### 2.4. Bagging Procedure

**Figure 1:**
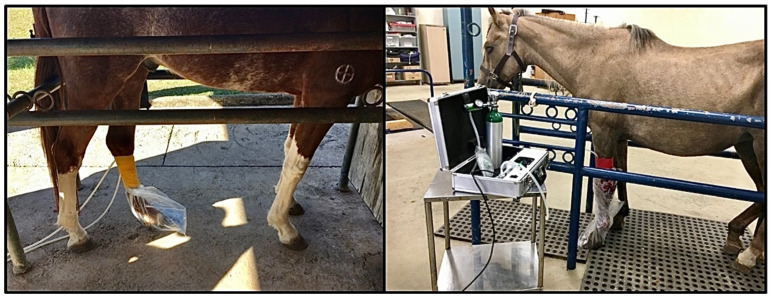
Ozone application—bagging technique.

The wound-bearing limb was enclosed in an ozone-resistant polyethylene bag, which was secured proximally and distally with torniquets. A perfusion tube inserted at the upper margin of the bag was connected to the generator. Using the generator’s built-in vacuum function, ambient air was evacuated from the bag before filling it with ozone at a therapeutic concentration of 50 μg/mL, at normal atmospheric pressure, until full expansion of the bag (Figure 1). Each exposure lasted for 30 min and was repeated every 48 h until complete healing. After each session, residual ozone was safely removed using the device’s vacuum extraction and neutralization system. A sterile protective, non-adherent dressing with light compression was applied after each treatment to prevent environmental contamination of the wound and to minimize the development of exuberant granulation tissue. The dressings consisted of non-adherent microfiber gauzes, a thin layer of orthopedic cotton cast padding and a light compressive layer of cohesive bandage. Wounds were lavaged with sterile saline only to avoid interference with the therapeutic effects of ozone. No concurrent topical or systemic therapies were administered during ozone treatment.

### 2.5. Wound Measurement and Scoring

**Figure 2:**
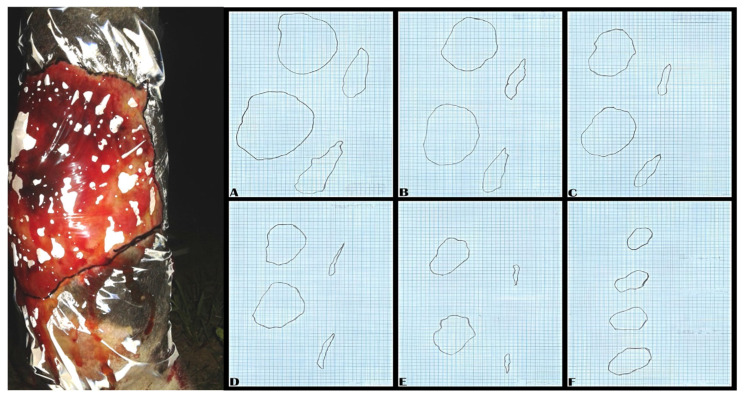
Wound perimetral printed on foil (**left**); prints in evolution reproduced on millimetric paper (**right A**–**F**).

Wound areas were recorded at each session by tracing their perimeters onto transparent plastic foil and transferring them to millimetric paper for surface measurement, as shown in Figure 2 [16]. The wound areas were expressed in square centimeters (cm^2^).

Due to variation in initial wound size, healing progression was also assessed using a four-grade epithelialization score adapted from previous wound evaluation systems [18]:

- 0 = no visible change;
- 1 = <50% reduction in initial wound area;
- 2 = >50% reduction in initial wound area;
- 3 = >75% reduction in initial wound area;
- 4 = 100% complete epithelialization.

All wound surface evolutions were scored independently by two equine surgeons for each timepoint (days 0, 14, 28, 44, and 58). After the millimetric paper surfaces were obtained, the differences were counted according to the initial timepoint (day 0) and scored accordingly, as described by the scoring system. Discrepancies were unlikely to appear considering the mathematical characteristic of the scoring system, but if they did occur, they were resolved by consensus.

Standardized photographic documentation was obtained from the same angle at each evaluation. Macroscopic assessment included the presence of scar tissue, the recovery of skin pigmentation and hair regrowth.

### 2.6. Statistical Analysis

Because the epithelialization score was an ordinal variable and the sample size was small (*n* = 9 lesions treated), data distribution was assessed using the Shapiro–Wilk test. Non-parametric statistics were used accordingly. Data are presented as median and interquartile range (IQR, 25th–75th percentiles). Differences in epithelialization scores across the five timepoints (days 0, 14, 28, 44, and 58) were evaluated with the Kruskal–Wallis test. When the Kruskal–Wallis test was significant, pairwise post hoc comparisons were performed using the Dunn and Mann–Whitney U tests with Bonferroni correction for multiple comparisons (10 total pairwise tests). Statistical analyses were conducted using GraphPad Prism 5.0 and Python 3.11, and significance was set at *p* < 0.05 (95% confidence interval) after adjustment.

## 3. Results

### 3.1. Description of Study Population

Before referral, all horses received prior field treatments for 1–7 months with combinations of topical antibiotics (oxytetracycline, neomycin, penicillin, streptomycin), systemic antibiotics, anti-inflammatory drugs, protective bandaging, and standard cleansing or debridement as required. Despite these interventions, the wounds did not show any satisfactory improvement. Unfortunately, detailed information about the previous treatments was unavailable as the owners did not have any files or medical letters for them.

Seven horses had primary chronic distal limb wounds, while one horse presented a large exuberant granulation tissue mass at the level of the right hock and had undergone repeated surgical excisions without favorable healing before referral.

**Figure 3:**
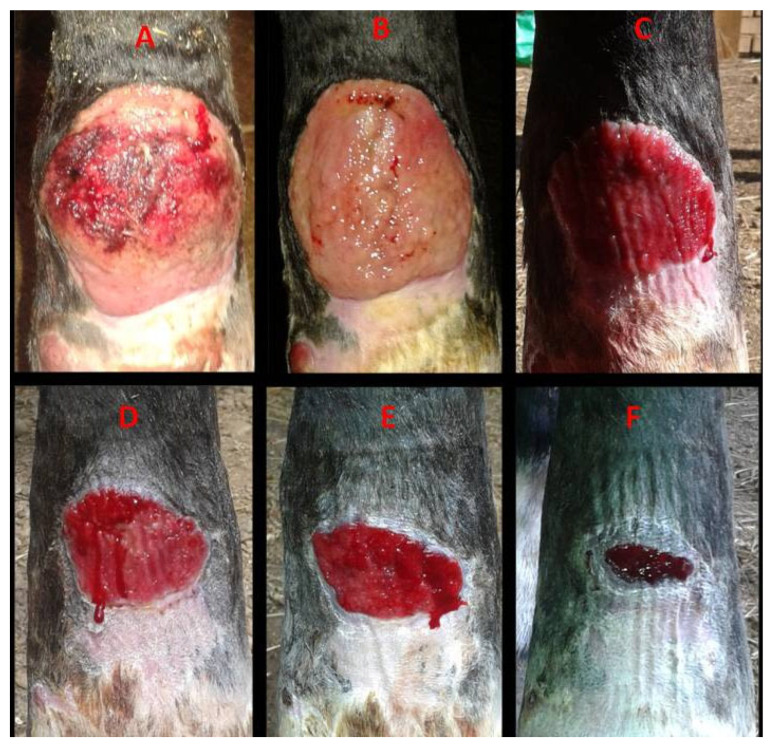
Case 1. Healing progression of a hypertrophic dorsal fetlock wound on the left forelimb: (**A**)—day 0: atonic wound with exuberant granulation tissue with superficial necrosis; (**B**)—day 7, reduction in granulation tissue with early contraction; (**C**)—day 14, leveled granulation tissue with initial epithelialization; (**D**)—day 28, progressing epithelialization; (**E**)—day 36, continued epithelial advancement; (**F**)—day 44, advanced epithelialization with a marked reduction in wound area.

**Figure 4:**
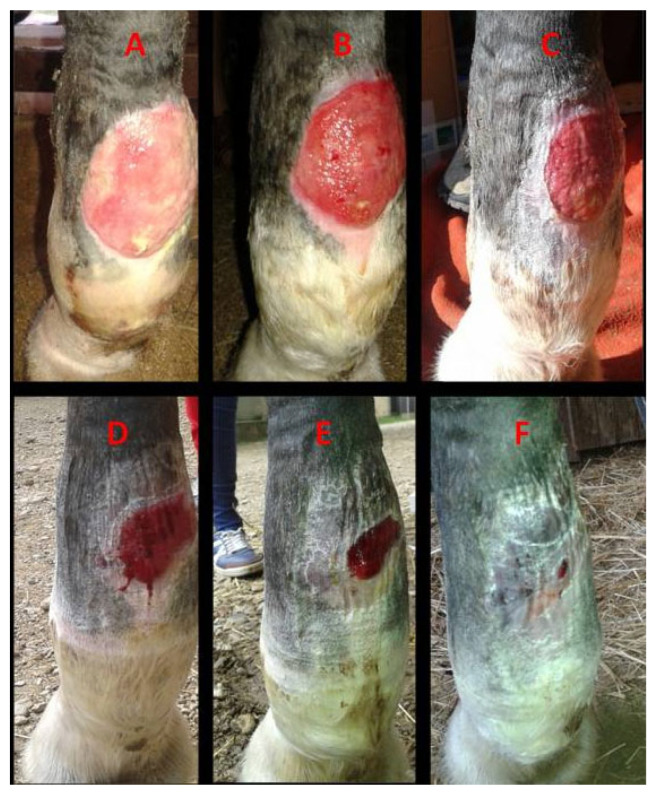
Case 2. A macroscopic evaluation of a chronic circular atonic wound on the palmar aspect of the left forelimb fetlock: (**A**)—day 0, exuberant granulation tissue with superficial necrotic debris; (**B**)—day 7, reduced granulation tissue and onset of contraction; (**C**)—day 14, leveled granulation tissue with early epithelialization; (**D**)—day 28, active epithelialization; (**E**)—day 36, continued epithelial progression; (**F**)—day 44, advanced epithelialization with near-complete surface coverage.

**Figure 5:**
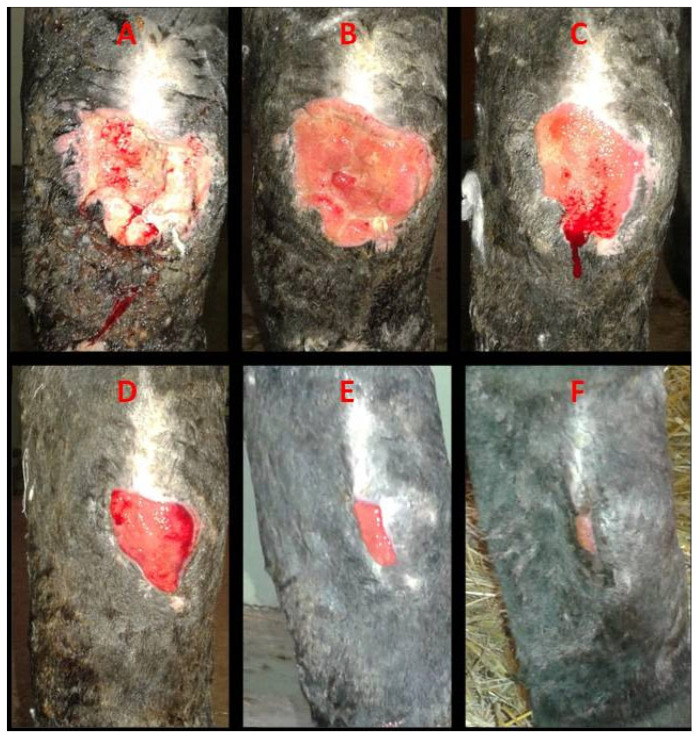
Case 5. Healing of a post-excisional chronic wound on the lateral aspect of the right hock following the removal of a hypertrophic mass: (**A**)—day 0, deep atonic wound with altered perilesional structures; (**B**)—day 7, active granulation; (**C**)—day 14, early contraction and epithelialization; (**D**)—day 28, ongoing contraction and epithelialization; (**E**)—day 58, advanced epithelialization; (**F**)—day 79, epithelialized surface with discrete residual scar tissue.

All eight horses completed this study. Complete wound closure was achieved in all cases, with healing times ranging from 27 days (Case 7) to 91 days (Case 5). The median healing time was 66 days (IQR: 52–69.5 days) resulting in an IQR of 17.5 days. In most horses, epithelialization accelerated notably (>50–75% wound surface reduction) after the fourth week of treatment. Five horses exhibited restored skin pigmentation and hair regrowth, while three developed small non-functional fibrous scars. The clinical characteristics of each patient, including breed, age, lesion type, location, and healing duration, are summarized in Table 1. Moreover, a selection of photographic evolutions representing cases 1, 2, and 5 are detailed in Figure 3, Figure 4 and Figure 5.

### 3.2. Epithelialization Score Analysis

Nine wounds were evaluated at five timepoints (day 0, 14, 28, 44 and 58) using a 0–4-grade epithelialization scale. Table 2 summarizes the results. Because score distribution was non-normal, a Kruskal–Wallis test was applied, revealing a highly significant difference across timepoints (*p* < 0.001). Post hoc pairwise comparisons showed that epithelialization scores at day 14, day 28, day 44, and day 58 were all significantly higher than baseline (day 0) after Bonferroni correction (adjusted *p* ≤ 0.0015).

**Figure 6:**
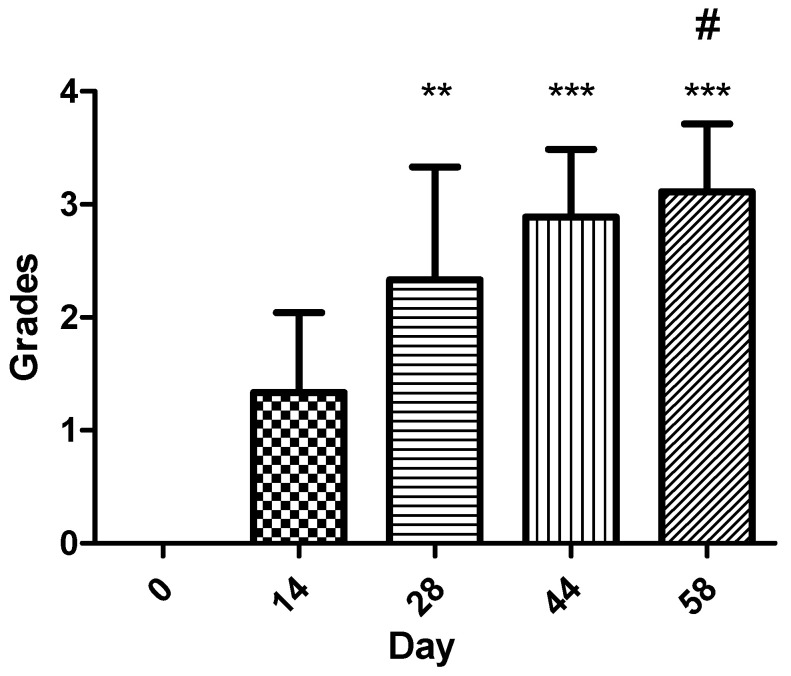
Epithelialization score progression across evaluation timepoints (*n* = 9). Statistical analysis was performed using the Kruskal–Wallis test, followed by the Dunn and Mann–Whitney U post hoc tests with Bonferroni correction: ** *p* < 0.01, *** *p* < 0.001 vs. day 0; # *p* < 0.05 vs. day 14. The most pronounced increases in epithelialization occurred after day 28, with the greatest improvement occurring between days 44 and 58.

Comparison between later timepoints demonstrated significant increases between day 14 and day 44 and between day 14 and day 58, while most other pairwise comparisons were not statistically significant after Bonferroni correction (Figure 6). These findings indicate that the largest, most statistically robust improvements in epithelialization occurred after day 28, with the most marked epithelial progression occurring between days 44 and 58.

## 4. Discussion

The findings of this uncontrolled case series indicate that ozone bagging therapy was associated with improved wound contraction and epithelialization in a group of equine chronic distal limb wounds that had previously failed to respond to conventional treatments. Because ozone at high concentrations may exert oxidative effects through hydrogen peroxide generation, its use in controlled medical concentrations is intended to induce only a mild transitory oxidative stimulus, promoting antioxidant responses without overwhelming the tissues’ oxidative balance [17].

Although microbiological analyses were not performed in the present study, ozone’s well documented antimicrobial activity may have contributed to a cleaner wound environment and reduced surface contamination. This assumption is in line with earlier clinical reports [18]. The healing times observed here (50–91 days) fall within or slightly below the ranges reported for other topical strategies such as ozonated oils or advanced wound dressings [11,19,20]. Several mechanisms have been proposed to explain ozone’s potential contribution to wound repair, including enhanced tissue oxygenation, modulation of local oxidative balance, and stimulation of angiogenesis and fibroblast activity [21,22,23]. These processes support granulation tissue formation and epithelial coverage [24,25]. Furthermore, experimental studies have linked ozone exposure to increased expression of key healing mediators such as transforming growth factor-β (TGF-β) and vascular endothelial growth factor (VEGF) [1,13,16,26,27], which help to regulate fibroblast proliferation and collagen synthesis. However, the extent to which these pathways operate similarly in equine wounds remains uncertain, as robust clinical evidence is still limited [28]

As observed in our study, the rate of epithelial progression appeared to depend on initial wound size, depth, and tissue condition—factors widely recognized to influence equine wound healing [13]. Appropriate wound bed preparation including debridement of necrotic tissues at initial presentation and maintenance of cleanliness likely contributed to treatment success. The use of a protective–compressive dressing after each ozone session also played an important role by reducing contamination and preventing exuberant granulation tissue formation [29]. The necessity of the dressing warrants specific discussion, as compression is a known method for preventing exuberant granulation tissue in equine distal limb wounds. However, we consider ozone bagging therapy to have been an effective therapeutic agent, rather than merely the compressive bandage. Critically, all included cases were refractory chronic wounds that had failed to heal for one to seven months despite prior conventional management, which would typically involve some form of local treatment and bandaging. This failure suggests that compression alone was insufficient to heal the wounds. Furthermore, the observed outcomes, including complete healing, restoration of skin pigmentation and hair regrowth in most horses, and minimal scarring, reflect significant tissue regeneration. This regenerative outcome aligns with ozone’s documented effects at the tissue level. Therefore, while the compressive dressing played an essential supportive role in preventing contamination and mechanically stabilizing the region and the developing tissue, the overall success and accelerated healing of these previously stalled lesions point to the topical ozone treatment as the primary therapeutic driver. Future controlled studies are needed to precisely differentiate the synergistic contributions of ozone’s regenerative properties and the mechanical effects of dressing compression.

Evidence from other species supports most of these proposed mechanisms. Topical ozone has been shown to enhance healing in experimentally induced oral wounds in rats through improved angiogenesis and fibroblast proliferation [30]. Ozonated liquids may suppress nuclear factor kappa B (NF-kB) activity, suggesting anti-inflammatory and immunomodulatory roles [25,31]. Nitric oxide known to influence collagen synthesis and fibroblast function has also been implicated in ozone-associated wound responses [31,32,33,34].

Nonetheless, the complete mechanism of action of ozone in equine tissue remains insufficiently understood and requires targeted investigation.

In this small clinical series, wounds appeared to exhibit improved macroscopic vascularization during treatment, which may be related to ozone’s ability to stimulate angiogenesis, fibroblast activity, and collagen organization. Comparable benefits have been reported with other conventional and ozone-based approaches, although direct comparisons remain lacking [7,9,13,35,36,37,38,39]. For patient 7, an 18.5 cm^2^ chronic calcaneal wound achieved complete healing within 27 days (3 weeks and 6 days). When contrasted with the approximately 4-week epithelialization period reported for experimentally induced acute distal limb wounds of 3.14 cm^2^, the outcome indicates that the ozone bagging technique may have supported the resolution of a chronic lesion nearly six times larger within a comparable timeframe [2].

From a practical standpoint, ozone bagging therapy is relatively simple and economical to perform, with the primary cost being the ozone generator itself. Comparable positive outcomes have been described with ozonated sunflower seed oil which has shown efficacy in reducing hypergranulation tissue and supporting wound contraction in horses [23]. Nevertheless, further controlled comparative studies are necessary to assess whether ozone therapy carried out by bagging offers equivalent or superior outcomes to ozonated oil-based treatments.

Based on our clinical experience, ozone bagging may be implemented using an ozone-resistant plastic bag and a concentration of 50 µg/mL for 30 min every 48 h, followed by appropriate protective dressings. Current guidelines emphasize the importance of using ozone-compatible materials and maintaining wound hygiene to support predictable outcomes [10]. Such preliminary recommendations provide a practical framework for clinicians until standardized protocols are further validated.

Beyond chronic wounds, ozone therapy may prove useful in other equine applications, including the management of post-surgical wounds or infected lesions or the prevention of exuberant granulation tissue in high-risk cases [23,40]. However, evidence supporting these uses remains limited to isolated reports.

This study is a case series and has several limitations. First, the absence of a control group or comparison to other standard therapies prevents any causal inference regarding ozone’s effectiveness. Second, the sample size was small (eight horses; nine lesions), and the cases were heterogeneous in terms of wound sizes, location, prior treatments, immune status, and age. Third, the lack of histological, immunohistochemical, or microbiological evaluation precludes insights into the cellular and molecular mechanisms underlying ozone’s beneficial effects, especially fibroblast proliferation, angiogenesis, and growth factor expression. Tissue biopsies and quantitative bacterial assessments would have strengthened the conclusions and helped to differentiate ozone’s antimicrobial and pro-healing effects. Further equine studies should incorporate tissue biopsies and serological assays to validate whether similar pathways, including growth factor activation, are engaged in horses, thereby elucidating ozone’s mechanistic role in equine wound healing.

Although all horses demonstrated complete healing and most showed restoration of pigmentation and hair growth, these outcomes should be interpreted cautiously given to this study’s observational design.

The prior failure of conventional therapies suggests that ozone may offer a viable approach in refractory cases; however, definitive conclusions require controlled, prospectively designed trials. Silva et al. (2024), in a review on ozone therapy in equines, has mentioned only one case report using the ozone bagging method [40]; considering this, according to our knowledge, our study is the first short case series where the ozone bagging method is used for the treatment of chronic limb wounds in horses.

Even if, according to the presented results, ozone used alone in the treatment of equine chronic distal limb wounds may cover the need for complete healing, this hypothesis needs further research. The positive results obtained at the end of this clinical research encourages future studies, including those with multiple study groups and other relevant methods of evaluation and quantification of data, in order to obtain more homogeneous and relevant results. Making a comparison between ozone treatment methods and other conventional treatments would be an interesting perspective for future studies.

## 5. Conclusions

In this case series, topical ozone therapy administered by the bagging method was associated with complete healing of chronic distal limb wounds in horses that failed to respond to conventional treatments. Healing times ranged from 50 to 91 days, and most horses demonstrated satisfactory skin remodeling with minimal scarring. While these outcomes are encouraging, they must be interpreted cautiously. This study lacked a control group and did not include histological or microbiological evaluations; therefore, causal effects cannot be inferred. The present findings provide only preliminary observational evidence suggesting that ozone therapy may represent a useful complementary option for managing refractory chronic equine wounds. Further research should include controlled, randomized trials to validate the therapeutic contribution of ozone and to compare different ozone delivery methods with current standardized treatment protocols. Additionally, the intricate nature of the ozone positive effect in equine wound healing might be further revealed by immunohistochemical and serological analyses in more homogeneous wound models, helping to clarify the mechanisms through which ozone may influence angiogenesis, fibroblast activity, and tissue remodeling in equine wound healing.

## Figures and Tables

**Table 1:** The clinical presentation of the cases.

Case	Breed	Age (Years)	Lesion Site and Aspect	Initial Wound Size (cm^2^)	Time to Heal	Results
1	Selle Francais mare (Figure 3)	16	Hypertrophic wound at the dorsal level of the anterior left fetlock	57.5	54 days	Normal tissue, pigmentation, and hair regrowth
2	Selle Francais mare (Figure 4)	17	Chronic circular atonic wound on the palmar aspect of the left forelimb fetlock	73	50 days	Normal tissue, pigmentation, and hair regrowth
3	American Pinto mare	10-	Elliptic chronic atonic wound on the lateral aspect of left forelimb fetlock	21.5	61 days	4 cm^2^ central fibrous scar
4	Lipizzaner mare	21	Supracoronary chronic wound on the medial aspect of the left hindlimb	74	67 days	4 cm^2^ central fibrous scar
5	Arabian stallion (Figure 5)	3-	Chronic wound after surgical removal of a hypertrophic mass the lateral aspect of the right hock.	37.5	91 days	Discrete scar; pigmentation and hair on 90% of the surface
6	Romanian sports horse	7	Multiple wounds on left forelimb fetlock	27.7	66 days	Normal tissue, pigmentation, and hair regrowth
7	Frisian stallion	3	Two wounds on the lateral aspect of hock (62.7) and caudal calcaneus (18.5)	62.7 18.5	66 days 27 days	Normal tissue, pigmentation, and hair regrowth
8	Lipizzaner mare	9	Chronic atonic wound at the right forelimb fetlock	66.7	72 days	6 cm^2^ fibrous scar tissue

**Table 2:** Epithelialization scores (0–4) across evaluation timepoints.

Wound Number	Case Number	D0	D14	D28	D44	D58
**1**	1	0	1	2	3	3
**2**	2	0	2	3	3	4
**3**	3	0	1	1	2	3
**4**	4	0	1	2	3	3
**5**	5	0	1	1	2	2
**6**	6	0	1	2	3	3
**7**	7	0	1	3	3	3
**8**	7	0	3	4	4	4
**9**	8	0	1	3	3	3
**Median**		0	1	2	3	3
**(IQR)**		(0-0)	(1-1)	(2-3)	(3-3)	(3-3)

## Data Availability

The original contributions presented in this study are included in this article. Further inquiries can be directed to the corresponding author.
